# Medical Reform

**Published:** 1847-11

**Authors:** W. L. Sutton

**Affiliations:** Georgetown, Kentucky


Art. III.—*Medical Reform. Reflections growing out of the Action of the Nationul Convention of* 1847. By W. L. Sutton, M.D., of Georgetown, Kentucky.

It is generally known that a movement is now being made by the medical profession, the object of which is to increase the respectability and usefulness of that profession. As this movement is calculated to exert considerable influence upon the public health either for good or evil, it is the duty of every member of society to give the subject a due degree of consideration. To the members of the medical profession, as conservators of the public health, will perhaps be awarded the duty of conducting this matter to its final consummation. It is therefore the duty of the physicians through the length and breadth of the land to consider the matter well, and give each his influence to keep things in their present state, or to make such alterations as may be deemed best calculated to promote the public good.

We know that two years ago a proposition was started to hold a national medical convention, one object of which should be to promote a more extended education, both scholastic and professional, than many graduates at present possess. This proposition was scarcely made, before Professor Payne, of the University of New York, took the field against it. The convention met, however, in the city of New York, in May, 1846. As soon as the convention was organized, a resolution was introduced by Dr. Bedford, and seconded by Dr. Pattison, both colleagues of Dr. Payne, declaring the attempt to form a national convention a failure, and proposing an adjournment sine die. To the honor of that convention, it is recorded that the mover and his second were the only yeas upon the motion.

The convention raised committees upon various subjects, among which was one on the preliminary studies of students, one on the professional education of students, and one on the propriety of separating the power of conferring degrees from the teachers of medical schools. Upon these three subjects we are to have a contest. I say upon these three, because—although the convention which met at Philadelphia in May last, with considerable unanimity agreed upon the requirement to be exacted of medical students as to preliminary education, and upon some recommendations as to their instruction at medical schools, leaving the subject of separating the business of teaching and licensing for consideration in 1848—yet scarcely had Prof. Annan, of the Transylvania school, arrived at home, before he set about nullifying the action of the convention of which he had been a member. This paper appeared in an extra of the Western Lancet, edited by another professor of the same school, prefixed by the following paragraph: "In place of copying the minutes of the convention, we insert the following paper, written by Prof. Annan, who was a member. It embodies, we are of opinion, very nearly the opinion of the profession here." This extra was extensively circulated.

Upon this introductory paragraph two remarks very obviously present themselves to my mind:

1. Inasmuch as the paper is a direct attack upon the wisdom of the measures advised by the convention, common courtesy, and, I may add, fairness, should have suggested the propriety of permitting that convention to be heard through its own acts. By this course there would have been little danger of those acts being misunderstood.

2. If this paper embodies the sentiments of the profession about Lexington, I apprehend there are few other places in which the sentiments of the profession correspond with it.

With these preliminary observations, I shall proceed to express my own views upon the subject of medical education, during which I shall occasionally refer to Prof. Annan's paper, as I presume it occupies the ground common to those who oppose the measures advised by the national convention. It is, in fact, the only one I have seen.

In all matters of controversy, it is important that the point in dispute be distinctly stated, and that collateral matters be kept from occupying more than their proper space. The matter in hand is not whether we have too many physicians—

not whether the old or the young physician is entitled to preference—not whether we should have a few or many medical schools—not whether the facilities for imparting medical education are greater now than they were formerly—not whether men would practice medicine without a diploma—not whether many gentlemen educated at the present time are not what their diplomas declare them to be, gentlemen and scholars, and entitled to the fullest confidence of the community; but it is, would the profession as a body be more respectable—would the interests of the community to which they minister be promoted—by requiring a more elevated standard of education, both academical and professional, in those who are declared by our licensing boards to be qualified to take charge of the health and lives of their fellow men?

Such being the question at issue, let us ascertain what the convention recommended. Prof. Annan says (Extra, p. 7): "The recommendation is, that no young man be permitted to enter the office of a physician, as a student of medicine, who is not acquainted with the Latin and Greek languages, and with the elementary mathematical sciences, including geometry and algebra, and also with natural philosophy; and that the medical colleges allow only such to attend their lectures and obtain degrees."

Again: "If all who obtain the degree of Bachelor of Arts from literary institutions should study medicine, they would scarcely supply the demand for physicians." A fair interpretation of this would be, that the convention advised that none but Bachelors of Arts should be permitted to enter the profession.

What said the convention? "That this convention earnestly recommends to members of the medical profession throughout the United States to satisfy themselves, either by personal inquiry or the certificates of competent persons, before receiving young men into their offices as students, that they are of good moral character, and that they have acquired a good English education, a knowledge of natural philosophy, and the elementary mathematical sciences, including geometry and algebra, and such an acquaintance at least with the Latin and Greek languages as will enable them to appreciate the technical language of medicine, and read and write prescriptions."

I remark here, that the professor must be supposed to have written his version of this recommendation without the benefit of having the proceedings of the convention before him. And further, this instance confirms the caution mentioned with regard to the introductory paragraph of the extra, because it seems to me that the two phases make very different impressions upon most minds—the one being an evidence that its possessor had passed a regular collegiate course of study; the other, that he had a little more learning than is necessary to enter college. In judging of the acts of the convention, we will in justice take its own expression of opinion as the criterion as to what is meant.

Here, as a loop upon which to hang a protest, I will quote a sentiment expressed on page 8: "It is not thought to be a difficult matter to acquire a sufficient knowledge of the various maladies to which human beings are liable, to qualify a man to become a physician." Although this is a form of expression which men frequently use to express their own opinions, I cannot believe Prof. Annan used it for that purpose, notwithstanding he expresses no dissent from it. After a period of thirty-two years spent in the profession, with the advantage of some previous training, I am constrained to say that I still find myself placed in circumstances in which the most solicitous attention is required to enable me to chalk out my course. Nay, after the most anxious reflection, I occasionally regret that I had not adopted a course more or less different. Such I believe to be the experience of those who properly appreciate their responsibility to their patients, to the community, and to their God. How often do we find instances of what would seem to be palpable mistakes in diagnosis, made by the most illustrious men in the profession! And shall we say that it is not difficult to acquire sufficient knowledge to qualify us to practice medicine? If, then, it is difficult to qualify ourselves to practice our profession, it follows that some training is necessary. As our profession is one which emphatically requires strength and accuracy in the reasoning powers, it would seem that such studies as invigorate these, and give us precision of ideas, are best calculated to answer that end. But the mathematical sciences are prëeminently calculated to sharpen our powers of investigation, and give us precise ideas; and therefore the convention has done well in recommending such a course of previous training, as one step towards fitting young men to undertake the study of medicine.

Again, it is to be hoped that no one's notion of 'free trade' goes so far as to advise that a young man, who is unable to read, should be encouraged or even permitted to study medicine. If so, then there is some limit, and to a certain extent a *monopoly.* But what is included in the word '*read*'? Is it the ability to pronounce words as spelled by certain letters? or does it imply the understanding of the ideas intended by the writer of those words and sentences? Most certainly the understanding of ideas is implied. Then to read a medical book implies to understand what is therein contained. Assuredly, then, a student of medicine ought to have such a knowledge of Latin and Greek as would enable him to appreciate the technical language of his profession, and read and write prescriptions. If he has not that knowledge, how is he to know that he attaches proper or even analogous ideas to the words he reads? Take an example: Some years ago a friend of mine, a graduate of a respectable medical school, came across the term *hydrargyrum cum creta.* He was very much at a loss to know what it meant, saying that he knew that 'hydrargyrum' meant mercury, and 'creta' meant chalk, but he could not find such a medicine as *cum* in the whole materia medica.

But how did the idea that it is not a very difficult matter to acquire sufficient knowledge to practice medicine, ever become common? I presume just in this way: the community found men in the profession with and without much education, some of whom, it was known to all, had had very limited opportunities for acquiring information; they found them equally armed with diplomas, the least educated taking most pains to exhibit them, and also speaking with most confidence of their ability to cure disease. In this state of things, it was entirely natural that the community should conclude that much knowledge was not necessary. But who is to blame for this? Not that community which had no correct means of forming an opinion; but those who, being judges, conferred the honors of the doctorate upon those utterly unfit to receive them.

But we are told (p. 7) that "this plan begins at the wrong end. The community must first receive a liberal educaation, and then there will be a demand for at least an equal degree of attainment among physicians." Has it come to this? Is it possible that a teacher in a respectable medical school should be willing to see the day when that profession of which he is one of the dignitaries—that profession of which it was formerly said that it contained more learning than either of the other learned professions—should be content to follow in the wake of *the community?*—that the time should actually come when a physician should be *required* to have an education "at least equal" to the community in which he lives? This would be beginning "at the wrong end" with a vengeance! Think you that those physicians who have done so much to ennoble our profession and the human race—men who reared those splendid edifices of which the professor speaks with laudable pride, whom he considers samples of the profession as it is, but whom I should rather consider samples of the profession as it has been—were men who "could not find such a medicine as *cum* in the whole materia medica," or that their "learning as a general rule is in correspondence with the state of general information among the people?" I tell you nay; they were men who were educated above their fellows, and prided themselves in their profession.

Again, arguing against the necessity of extended education, he says (p. 8): "On the contrary, we maintain the opinion that young men of plain education, who have had their constitutions invigorated by labor from their youth to manhood, are the best qualified for these situations," (rude sections of country.) "The course of study which such young men pass through, while preparing themselves to graduate at one of our medical colleges, will expand and strengthen their minds in at least as great a degree as any course of Latin and Greek and mathematics of the same duration. And when we consider that one great object of education is to develop the mental faculties, it must be obvious that if this is effected by the study of medicine, while at the same time knowledge is acquired which prepares for usefulness as a practitioner, all this outcry about the absolute necessity of a thorough preliminary education is supremely ridiculous."

I shall not labor to prove that the study of medicine does not improve the mind—no, nor the heart; but I should dislike to be required to prove that because a perennial plant brings forth seed in the third year, therefore the growth of the two previous years was unnecessary. Neither shall I attempt to prove that *if*—"If is your only peacemaker"—we can reap a crop while clearing the ground, all the ado about previous clearing and cultivating "is supremely ridiculous." Let me put a plain question or two. What time is necessary for a Bachelor of Arts to acquire such a knowledge of medicine as to justify his engaging in practice? Will any one say less than two years? I should like to see the man, who knows anything of the profession, who would. How long would it require a young man who has very little education, not accustomed to exercise his reasoning powers upon intricate subjects—one "invigorated by labor from youth to manhood"—to master the same amount of medical information which the A.B. would acquire in two years?

But another difficulty, "*insuperable difficulty*," is, "physicians engaged in an extensive practice hold out inducements to the youth in their respective neighborhoods to enter their offices as students, because it relieves them from a great deal of labor," and "the ordinary fee is not demanded." This is all true, but not all the truth. *No instruction is imparted.* But it is becoming even worse than the professor is aware. Men begin to think that their sons ought to receive compensation from the physician, because of the services which the pupil renders his preceptor—*preceptor?* forgive the mistake—the physician. With no preparatory training or habits of reflection—left to pursue pretty much his own course of reading such books as he can get—with no examinations or instructions from the physician—how can the student be expected to profit much by hearing six or seven lectures, upon as many different subjects, daily, without time to reflect upon one before the delivery of another? I do not attempt to remove any of the blame which justly attaches to this reprehensible conduct on the part of the practitioner; but I may be permitted to ask, would he dare to pursue this course if he believed that his protegé would be subjected to an examination calculated to test his acquirements?

It is said, further, that under the course advised by the convention, "thousands of practitioners of medicine would spring up, whose only sources of information would be a little reading at their own homes; and thus in a few years we would have a host of doctors entirely ignorant of anatomy, chemistry, pathology and surgery, and also of therapeutics until repeated and fatal experiments upon their fellow creatures should have enabled them to discriminate disease, and apply the proper treatment." I would ask, has the professor any objection to this exemplification of 'free trade?' It certainly is only carrying out the general expression of sentiment contained in his paper. But I hope and believe better things of him. But whether he objects or not, we nave just that class of practitioners, not, however—at least not all of them—with information acquired "by a little reading at their own homes," but with that which, if not acquired at college, such as they possessed after leaving college.

For example, I have heard one gentleman talk about the prostate gland in the female. I knew one who applied a tight bandage above a wound of the femoral vein, and suffered his patient to bleed to death. I heard one inquire whether the yellow ground of labels for shop furniture was not imparted by aqua fortis. Ay, I heard a professor in a medical schoo propose a prescription composed of *calomel and carbonate of ammonia.* Let me close this list by giving a portion of a conversation between an eminent professor and a gentleman who went some distance to consult him: The professor asked the patient what was the opinion of his family physician as to the disease. Upon being told, he replied, "He is a most egregious quack." To which the gentleman replied, "I reckon not, doctor; at any rate he has your certificate that he is qualified to practice."

I understand the intention of the convention to have been, not to prevent the unqualified from practicing, but to compel them, if they will do so, to stand upon their own merits, not to be countenanced, aided and abetted by respectable men. Suffer me to put a case, and charge me not with disrespect; I certainly feel none: Two men go into a community where they are wholly unknown, one without any introduction or recommendation, the other highly recommended by six or eight of the most prominent and respectable men say of Lexington or Philadelphia. After a residence of some time, it is ascertained that both of them are swindlers and pickpockets. Which will have been enabled and which will probably have committed the most extensive depredations? I hold there is but little difference between this case and that of two unqualified physicians, one armed with a diploma, the other not; in one case we lose our property and money, in the other our health and lives. The physicians may be thought uninfluenced by felonious motives—may have no malice towards any member of the community. The same may be supposed of a man who shoots into a crowd; he may have malice against no individual; he may be supposed not to wish to hurt any one. But the law holds such a man to be either a felon or a lunatic; so should that man be held who practices medicine without qualifications.

It is our duty to uphold the respectability of the profession. This can only be done by keeping the great body of its members individually respectable. Men generally are badly qualified to judge of the attainments of physicians. The best criterion which they have is to compare their information upon matters of general knowledge with that of well instructed members of the community, and estimate their professional standing accordingly. I have known physicians tested in this way, and have heard very ludicrous as well as humiliating remarks as to the results of such trials. How would a physician whose prescriptions afford such specimens as this—" Take fifteen drops of *lodame acashunally*"—be estimated by this test? Yet the man who wrote that sentence, which is a sample extracted from about half a page of foolscap, claims to be a graduate

" It is a melancholy truth, that some of the students of medicine attend our medical colleges more for the purpose of obtaining the honor supposed to be conferred by the degree of Doctor of Medicine, than to acquire the requisite qualifications for becoming skillful physicians. This will invariably happen where a high standard of attainment is not required by the people of any particular district." [Ought not the professor to have added, " Nor by the teachers in our medical schools"?] " The title of doctor is sufficient to give rank among an unenlightened community."

Take this quotation, what is expressed and what is directly implied, and it contains the strongest opprobrium on our profession which I have ever seen conveyed in the same number of words. In the first place, the honor attached to the degree of M.D. is fictitious, not real; next, it is understood that this honor can be acquired without the knowledge which the possession of the degree implies; and thirdly, the title is sufficient to give rank among an unenlightened community. The time was when the degree of M.D. was a real honor, and conferred rank anywhere. Is it an understanding that our colleges will confer the degree for so much money? Do they, like other factories, produce wares of different values but of the same name, to suit various markets? If so, is the price graduated according to the quality? If none of these questions are to be answered in the affirmative, why do these men receive the doctorate?

The convention recommends also that physicians shall give to their students certificates as to their academical studies; and also the time at which they commenced their professional studies; and further, that colleges should exact such certificates. We have no means to judge what respect will be paid to that part which relates to the commencement of studies. Even if that should be observed, something would be gained; for although our schools generally state as a requisite that three years' pupilage should be observed, yet no means have been used to enforce this rule, and I presume no inquiry is ever made upon the subject, as young men are graduated whose pupilage has not continued half that time.

As an objection to a requisition of extended courses of lectures, it is urged that in some colleges a preparatory course is given without additional expense; but that few pupils avail themselves of the facility thus offered, or of attending additional courses after the second. I do not conceive the objection valid. In the first place, an attendance is not made necessary to graduation, and men are still disposed to get things with as little trouble as possible; secondly, it is usual, I think, for this preliminary course to be confined to some two or three professors, which pupils consider, whether wisely or not, an insufficient inducement to incur the additional expense of boarding.

But we are told, and told truly, that the profession is popular with young men. It is so with two very different classes of young men—those who are ambitious to spend lives of honor and usefulness, and those who are too lazy to work, yet wish to be respected. All of both classes who are worth encouragement will submit cheerfully to any reasonable course of study which respectable colleges will require, though they may not incur any avoidable expense, for most students have no great redundance of money. When an exalted standard is fully recognised, I doubt not that many young men of promise will engage in the profession, who will not do so now, because of the great competition, knowing that young men of no education leave college upon a perfectly equal footing with those of the most finished.

There is one sentence in the paper so often quoted, which I confess made me open my eyes; it is this: "In all parts of Europe the medical profession is much more crowded than with us." Although this is a matter, as I conceive, altogether irrelevant to the question at issue, yet as it seems to be urged as a justification of the course pursued in this country, I will remark that I do not know what data the professor has by which he arrives at his conclusion; certainly it is precisely the reverse of my impressions upon that point. I find, too, that the committee on medical education, who may safely be supposed to be better informed than either Prof. A. or myself upon this subject, speak of the physicians of the United States being far more numerous in proportion to the population than in any other country, perhaps, of which we have any correct knowledge. In France, with a population of 35,000,000, there are from 18,000 to 20,000 physicians, including officers of health, [*Boston Med. and Surg. Jour. July* 14, 1847,] or one to every 1750 inhabitants; in the United States, about 40,000 physicians to 20,000,000 inhabitants, or one to every 500. Again, in France there are 1800 medical students; in the United States, 4418; in France, from 300 to 700 graduate annually; in the United States, 1300. [*Id. May* 5. *Medical News*, *May*, 1847.] There is certainly no comparison between the amount of study required of a student here and in any of the nations of Europe.

Prof. Annan, in the paper referred to, and in one published in the September number of the Western Lancet, insists manfully that the convention is not entitled to the epithet 'national.' In support of his position he says: "I would now ask by what authority did the Medical Society of the State of New York call a convention? The ordinary mode of convening such assemblies is to take a vote of all those interested as to the necessity of such a meeting. It may therefore be said to savor strongly of presumption for any society or the societies of any State to issue proposals for a convention of representatives from the whole country." Men from beyond the mountains sometimes answer one question by asking another. I might then ask, how is a vote of the physicians scattered over these United States to be taken? Strongly as it savors of presumption, I see no way in which the object could have been obtained but that pursued. How are political conventions of States and of the Union got together? Some body of men, not more respectable than a State medical society, or even some individual proposes that a convention be held at a given time and place, for certain purposes. If the proposal meet with general approbation, delegates are appointed from divers portions of the country to attend. If the country embraced by the proposals generally sends delegates, it is legitimately called a district, State, or national convention, as the case may be, notwithstanding a small portion of the country may have failed to appoint delegates, or those delegates may have been prevented from attending. Why should not the same rule apply to the medical convention? In this case delegates from twenty-three States were in attendance. How many States appointed delegates who were unable to attend, I do not know; we know, however, that at least three corporations appointed delegates who failed. Upon this head I must be permitted to give my suffrage in favor of the grounds being sufficient to entitle the convention to the name "national."

Another reason why the convention is not entitled to the name is, that it was composed of one party to reform and one to be reformed, the second being the professors of medical schools. I am of opinion this division is rather arbitrary. I understand the convention to have been composed of one body to be reformed, and the same body to reform. It was admitted on all hands that there were evils to be remedied, which the profession must do or let go undone. Why it should be supposed that the medical schools alone were to be affected, I am at a loss to conjecture. Surely action was had on subjects intended to bear on individuals as members of the profession. Why, then, are not the individual members of the class to be reformed?

Another reason why it was not "national" is, that only a little more than one-fifth of the members of the convention were professors in medical schools. One might think that was a moderately fair proportion, when we consider that of the forty thousand physicians in the United States, something less than two hundred are engaged as teachers. From the considerations before mentioned, the professor concludes that "it was not a fair representation of the profession of the entire country." This may be true if it applies to the disproportion in representation of the parts near to and remote from the place of meeting; but I hope and believe that time will prove that the convention gave a fair exposition of the opinion of a large majority of the profession in the United States.

This paper has grown much beyond its contemplated limits, and although the points touched might be much amplified and many more introduced, yet as I hope to see an expression of opinion from divers quarters, I am disposed to close what I have to say upon this subject. In taking my leave of Prof. Annan's paper, I will remark that although its general tenor is such as to produce a deleterious influence in the profession, yet I am pleased to see occasional sentiments which are dictated by a commendable professional feeling. Indeed, the impression made upon my mind is as if the author felt himself called on to defend a course of the full propriety of which he is not entirely satisfied.

The resolution intended to separate the duties of teaching and licensing will no doubt give rise, at the next meeting of the association, to a very animated and interesting discussion. Indeed, it is time that the members of the profession were interchanging opinions upon the subject. It is one of momentous interest, and requires to be handled with much discretion. Neither ought its destiny to be left entirely in the hands of those who may compose the association in 1848. The length of this paper precludes the idea of my taking up the subject, even if I felt qualified to discuss it.

The convention performed a very important and much needed service in adopting a code of medical ethics. It is to be hoped every physician in the United States will procure a copy, and give it an occasional reading. Upon most subjects, perhaps contained in the code, the dictates of good breeding and gentlemanly feelings would always be a sufficient guide; but some regulations are purely conventional, and therefore require some recognised authority to insure their observance. It is hoped that a proper attention to this code will make professsional intercourse more pleasant to physicians and beneficial to patients.

One of the most important measures of the convention is that recommending the State legislatures to pass laws for keeping a register of marriages, births and deaths within their respective districts. It is impossible to know the extent of good which will result to the nation by a general adoption of this measure. From some little attention paid to this subject on a small scale, I am disposed to believe that our profession and the community will be very much startled by the developments which the execution of this measure will make. It is assuredly the only correct way of arriving at the relative salubrity of different sections of the country; and I hazard the opinion that, if carried out, some portions of country, the citizens of which congratulate themselves upon superior enjoyment of health, will be found to present a mortality altogether unlooked for. It is sincerely to be hoped that physicians generally will urge upon their immediate representatives the importance of this matter. As this measure is to promote the general good, and not that of the profession, there surely can be no objection to its adoption.

When the true state of health of a district is thus made manifest, and at the same time the diseases most prevalent there ascertained, the next step will be to determine the causes producing these diseases, and next the means of removing these causes, or neutralizing their deleterious influence.

Connected with registration, and essential to its proper execution, was a nomenclature of diseases adopted by the convention. A proper attention to this will enable us to state the causes of death in a more uniform and consistent form than is to be generally found in our bills of mortality.

Composed of representatives from twenty-three States, from twenty-eight medical colleges, sixteen State medical societies, besides various district medical societies, there is little room to doubt that this convention was as respectable as any body of men ever assembled in these United States. Neither do we doubt that this convention has exerted and will continue to exert a most beneficial influence upon the profession and upon the interests of the community, by embodying and directing public sentiment as well as the investigations upon medical subjects. It is true that individuals and corporations may attempt to decry and depreciate the authority, or rather the influence of this body for a while; but observing at last that this influence is not directed against any body or society which deserves countenance, they will fall into line, and be as much gratified as others that such an association was ever organized. As a physician, as a citizen, I hail this body as one calculated to do immense good, as the preserver of the dignity of the profession, which I considered endangered by what I conceived a suicidal policy adopted by many of our medical schools, and as calculated to promote the longevity, health and happiness of the community.

